# Space exploration and lifestyle medicine: a narrative review of its implications for astronaut health and remote Earth-based environments

**DOI:** 10.3389/fphys.2025.1729596

**Published:** 2026-01-13

**Authors:** Raphaëlle Giguère, Alexandre Marois, Daniel Fortin-Guichard, Jonathan Charest, Julie Rocheleau, Keith Thompson, Michael Stolberg, Philippe St-Martin, Audrey Bergouignan, Victor Niaussat, Jean-Sébastien Paquette, Marie-Pierre Gagnon, Maxime Sasseville, Caroline Rhéaume

**Affiliations:** 1 Department of Medicine, Faculty of Medicine, Université Laval, Québec, QC, Canada; 2 VITAM – Research Center on Sustainable Health, Québec, QC, Canada; 3 École de psychologie, Faculté des sciences sociales, Université Laval, Québec, QC, Canada; 4 Department of Kinesiology and Physical Education, Sylvan Adams Sports Science Institute, McGill University, Montréal, QC, Canada; 5 Idorsia Pharmaceuticals Canada, Montréal, QC, Canada; 6 Department of Psychology, Université du Québec à Montréal, Montréal, QC, Canada; 7 Department of Family Medicine, Schulich School of Medicine and Dentistry, Western Space Institute, Western University, London, ON, Canada; 8 Département des sciences de l’activité physique, Université du Québec à Montréal, Montréal, Canada; 9 Faculty of Physical Activity Sciences, Université de Sherbrooke, Sherbrooke, QC, Canada; 10 Research Centre on Aging, Université de Sherbrooke, Sherbrooke, QC, Canada; 11 Pluridisciplinary Hubert Curien Institute (IPHC UMR 7178), French National Center for Scientific Research (CNRS), Université de Strasbourg, Strasbourg, France; 12 Division of Endocrinology, Metabolism and Diabetes, Anschutz Health and Wellness Center, University of Colorado, Aurora, CO, United States; 13 Thales AVS, Mérignac, Nouvelle Aquitaine, France; 14 Primary Care Research and Innovation Laboratory (Laboratoire ARIMED), Groupe de Médecine de Famille Universitaire du Nord de Lanaudière, Joliette, QC, Canada; 15 Faculty of Nursing, Université Laval, Québec, QC, Canada; 16 Quebec Heart and Lung Institute-Research Center (IUCPQ), Québec, QC, Canada

**Keywords:** lifestyle medicine, microgravity, remote Earth-based environments, space exploration, space medicine

## Abstract

**Background:**

Space exploration, especially long-duration missions such as those to Mars, presents unique and significant challenges to astronaut health. Space medicine, which focuses on maintaining health in extreme environments without access to definitive medical care, emphasizes preventive measures. Lifestyle medicine (LM), grounded in six pillars such as healthy nutrition, regular physical activity, restorative sleep, stress management, positive social connections, and avoidance of risky substances, has proven effective for disease prevention on Earth. However, its application to spaceflight and remote Earth environments remains underexplored. This raises the question of how LM framework can sustain astronaut health and inform preventive and primary care strategies for remote Earth populations.

**Objective:**

This narrative review examines how LM can support astronaut health during long-duration missions and draws parallels with healthcare needs in remote Earth populations. It establishes principles for integrating lifestyle and space medicine and provides recommendations for their application in both contexts.

**Results:**

Each LM pillar is uniquely challenged in space. Nutritional constraints arise from limited food variety and storage capacity; microgravity and workload restrictions limit physical activity; circadian disruption and environmental noise affect sleep; isolation, confinement, and mission stress compromise stress regulation and social connections; restricted crew size and communication delays limit social connection; and strict medication policies highlight the dual role of substance use as both risks and necessity. While individual countermeasures have been tested in space, no integrated framework addressing all pillars simultaneously has yet been implemented. Technological innovations, such as wearable devices for continuous monitoring, telehealth modules for remote support, and virtual reality platforms for mental health and social engagement, appear as promising enablers of such an integrated approach for both astronauts and populations in medically underserved areas on Earth.

**Conclusion:**

LM provides a preventive framework that complements traditional countermeasure and may enhance resilience and autonomy during space missions. Future research should prioritize integrated, longitudinal studies in analog environments to quantify the synergistic effects of integrated LM interventions versus single pillar countermeasures. Its translation to remote and underserved populations on Earth could help reduce healthcare disparities and support scalable, autonomy-centered models of care, underscoring the bidirectional value of combining lifestyle and space medicine.

## 1 Introduction

Since the first human spaceflight in 1961 (NASA, 2024a), space exploration has raised fundamental questions about the safety and health of astronauts. As missions became longer, more complex, and more frequent, the need for specialized expertise led to the emergence of space medicine, a discipline dedicated to protecting health and optimizing human performance in space (Pool and Davis, 2007). Space medicine covers prevention, care, and performance optimization across all mission phases, from preflight screening to in-flight care and post-mission recovery (Pool and Davis, 2007). Space health must go beyond disease prevention to embrace a holistic approach that supports astronauts but also translates innovations to Earth-based populations facing similar challenges, particularly in remote and underserved Earth regions. By integrating diverse knowledge and addressing environmental concerns, space medicine can bridge technological progress with ethical responsibility (Canadian Space Agency, 2024).

On board the International Space Station (ISS), the crew currently has access to a wide variety of medical equipment, medication, and communication and consultation with the flight surgeon and ground crew (Smith et al., 2023). Space medicine and astronaut healthcare is an area where health management autonomy will become increasingly important, especially in the context of longer exploration missions to the Moon and Mars (Patel et al., 2020; Komorowski et al., 2021). Mission durations vary significantly, with short missions lasting less than 30 days, mid-term missions lasting up to 6 months on stations like Mir or ISS, and long-duration interplanetary missions presenting complex health challenges (Cinelli et al., 2020). Long-duration interplanetary and deep space exploration missions, like lunar bases and Mars colonization, present unique and extended health challenges (NASA, 2020; Air and Space, 2017; Kahn et al., 2014). These missions amplify the hostile space environment, requiring astronauts to adapt and to maintain physical health, cognitive function, and psychological resilience over prolonged periods. Recent studies have identified key physiological and clinical challenges associated with long-duration space missions and outlined directions for future research (Nguyen and Urquieta, 2023; Tomsia et al., 2024). One promising approach is lifestyle medicine (LM), a holistic, preventive model that emphasizes proactive health management that could offer evidence-based interventions to enhance resilience pre-flight, sustain health in-flight, and support recovery after missions.

The concept of LM emerged in the early 1980s, when cardiologist Dr. James Rippe first used the term to describe a medical approach for disease prevention and treatment through lifestyle interventions (Rippe, 2024). Since then, the field has evolved, with various organizations formalizing its principles. The American College of Lifestyle Medicine (ACLM), founded in 2004, played a key role in establishing LM as a recognized medical specialty (American College of Lifestyle Medicine, 2024). Other international organizations, such as the European Lifestyle Medicine Organization (The European Lifestyle Medicine Organization, 2025), the Lifestyle Medicine Global Alliance (Lifestyle Medicine Global Alliance - Global Vision of True Health Care, 2016), and the Australasian Society of Lifestyle Medicine (Egger et al., 2020), also contributed to its development. LM is grounded in six pillars: nutrition, physical activity, sleep, stress management, social connection, and avoidance of risky substances. It has been shown to prevent, treat, and sometimes reverse chronic disease (American College of Lifestyle Medicine, 2024; Paquette et al., 2023; Li et al., 2020). Together, these pillars form a conceptual framework, which can be adapted in extreme environments such as the unique challenges of spaceflight. While LM principles have been applied in healthcare contexts to prevent and manage disease (Lippman et al., 2024; Ornish et al., 1998), they also have broader, practical applications beyond traditional settings. For instance, an ongoing study on firefighters examined four of the six pillars to improve their health and resilience in high-risk occupations (Hershey et al., 2023). This naturally raises a broader question: How can the six pillars of LM be applied as preventive and supporting methods to sustain astronaut health during space missions, and what lessons can be translated to remote populations on Earth?

## 2 Objectives

The present narrative review explores how the space environment challenges the six pillars of LM and examines how LM could provide a holistic strategy to mitigate these effects. To the best of our knowledge, this is the first review to formalize an integrated LM perspective in this context, presenting a pillar-by-pillar synthesis while emphasizing their interdependence. The review seeks to provide recommendations for protecting astronaut health in future space missions, particularly in remote and resource-limited environments, while also generating insights applicable to Earth. More precisely, this review aims to: 1. synthesize current evidence at the intersection of space and LM; and 2. propose recommendations for reinforcing the six pillars before, during, and after space missions.

## 3 Methods

This narrative review synthesizes existing literature on the integration of LM into space exploration and its implications for astronaut health and remote Earth environments. The review was self-assessed using the Scale for Assessment of Narrative Review (SANRA) quality guidelines (Multimedia Appendix 1) (Baethge et al., 2019). A non-systematic narrative approach was employed, given the diversity of the domains and the wide range of journals and domains relevant to the subject (Foster, 2004). Peer-reviewed articles, reviews, and relevant grey literature were identified through electronic databases such as PubMed, Web of Science, and Scopus. Keywords included were “lifestyle medicine”, “astronaut health”, “space medicine”, “microgravity”, “space exploration”, “behavioral health”, “emergency space medicine”, “remote” and terms related to LM’s six pillars.

Expert opinions and reports from space agencies (e.g., National Aeronautics and Space Administration [NASA], Canadian Space Agency [CSA], European Space Agency [ESA]) were also reviewed to provide additional context. The synthesis outlines Earth-based recommendations, space-related constraints, potential applications, and proposed strategies for reinforcing each pillar before, during, and after space missions. Following the approach suggested by Kanas (2015), this review highlights opportunities for bidirectional knowledge transfer between terrestrial and space health contexts. Advances in space medicine can in fact have a positive impact on isolated terrestrial environments. Conversely, research conducted in preventive medicine on Earth can contribute to the development of health strategies tailored to the needs of astronauts. This allows common and adaptable mechanisms to be identified between the two contexts. To reach these goals, the framework of LM’s six pillars is applied systematically to examine astronaut health in the context of space exploration.

## 4 Six pillars of lifestyle medicine to support space exploration

### 4.1 Nutrition

Nutrition, as one of the six pillars of LM, employs wholefood, plant-predominant dietary patterns to optimize physiological function and reduce the risk of chronic disease (Kahleova et al., 2017; Kris-Etherton et al., 2001; Phillips et al., 2020; Satija et al., 2017). On Earth, clinical guidelines recommend consuming vegetables and fruits (≥5 servings/day) for vitamins, minerals, and phytochemicals; whole grains, nuts, and seeds to achieve 25–30 g of fiber; moderate protein from plant sources and lean animal products; and monounsaturated and polyunsaturated fats (e.g., olive oil, nuts) while limiting saturated and trans fats, ultra-processed foods, added sugars, excessive sodium, and alcohol (Cara et al., 2023; Hauser, 2022). Suboptimal nutrition is a major risk for noncommunicable diseases (Afshin et al., 2019; Forouzanfar et al., 2016; Murray et al., 2013): diets high in saturated fats, refined sugars, and sodium foster insulin resistance, hypertension, and endothelial dysfunction, key drivers of cardiometabolic disease (Mozaffarian, 2016), whereas high-fiber, antioxidant-rich, unsaturated fat and plant protein–focused diets alleviate low-grade inflammation and oxidative stress, mitigating risks of atherosclerosis, metabolic syndrome, and neurodegeneration (Satija et al., 2017). Plant-based (Barnard et al., 2006) and Mediterranean-style diets (Estruch et al., 2018; Nordmann et al., 2011) demonstrate broad health benefits (Katz and Meller, 2014). While it is difficult to determine a single optimal diet for health, LM integrates this evidence to support long-term behavioral changes that prevent, manage, or reverse diseases.

Historically, food availability and quality have been decisive for the success of expeditions and missions (Douglas et al., 2020). In spaceflight, nutrition ensures adequate intake to meet metabolic demands, sustain health, and support psychological wellbeing through the social value of meals (Bergouignan et al., 2016; Evert et al., 1992). Yet, space nutrition is constrained by physiological, logistical, and technological limits (Smith et al., 2019). Modern space food preservation (methods such as dehydration, thermal stabilization, and irradiation) ensures long-term shelf stability but often compromises nutrient retention and palatability (Cooper et al., 2011). A food system designed for long exploration missions, such as those to Mars and beyond, must reliably preserve sensory appeal, nutritional quality, and safety for a period of 3 to 5 years to be considered viable (Cooper et al., 2011). Analyses of space food, at both post-processing and after 3 years of storage at room temperature, have shown that essential micronutrients, such as potassium, calcium, vitamin D, and vitamin K, may already fall short of recommended daily intake levels even before storage begins (Cooper et al., 2017). Inadequate micronutrient content can impair immune function and antioxidant defense mechanisms, critical concerns for long-duration missions beyond low Earth orbit. Additionally, the absence of refrigeration, limited cooking options, and strict packaging requirements further limit the variety and palatability of available foods (Evert et al., 1992). Over time, menu fatigue and lack of fresh produce may decrease energy intake and contribute to micronutrient deficiencies.

Appetite suppression caused by the microgravity environment, potentially exacerbated by intensive exercise regimens, combined with altered taste and smell perceptions in space can lead to insufficient caloric intake and inadequate consumption of essential macro- and micronutrients (Laurens et al., 2019). However, further research is needed in order to identify the specific changes in taste and smell to prevent anorexia in space (Tomsia et al., 2024; Taylor et al., 2020), and to explore the relationship between nutrition and the other pillars in space. While a shortfall in energy intake may be tolerable during shorter missions aboard the ISS due to the body’s ability to draw on fat reserves, it poses significant risks for longer-duration missions. Together, these factors highlight the importance of developing nutritional strategies that guarantee sufficient energy intake, maintain nutrient balance, and support metabolic health throughout space missions. Six key nutritional challenges have been identified that are priorities for space missions: maintaining energy balance during flight; changes in eating behavior; the onset of metabolic stress; risk of micronutrient deficiencies; disruptions in gut microbiota; and imbalances in fluid and electrolyte levels (Bergouignan et al., 2016).

Building on these challenges, recent findings from 6-month missions aboard the ISS offer, for the first time in long-duration spaceflight, valuable insights into the consequences of these nutritional and physiological stresses. Bourdier et al. reported large individual variability in body composition during 6-month ISS missions, linked to changes in total daily energy expenditure measured by doubly labelled water (Bourdier et al., 2022). Astronauts who maintained preflight energy expenditure preserved lean mass and lost fat, while those with reduced expenditure lost lean mass and gained fat. In addition, mission-related stress and sleep disruption, both common in long-duration spaceflight, may further exacerbate appetite suppression and alter dietary intake patterns, highlighting the interdependence between nutrition and other LM pillars. These differences were associated with preflight fitness and exercise intensity. Standard aerobic exercise protocols in microgravity may thus contribute to chronic negative energy balance and health risks for long missions. In summary, some astronauts burn more than they eat and lose fat, while others burn less and lose muscle (lean mass).

This issue is further complicated by observed changes in dietary intake during spaceflight, including a shift in macronutrient consumption favouring carbohydrates over lipids (Le Roux et al., 2024). Despite some spontaneous adaptations, an uncoupling between energy intake and expenditure was observed in microgravity, contributing to a sustained energy imbalance during spaceflight (Bourdier et al., 2022). The cause of dietary changes in microgravity remains unclear, with potential factors including limited food options, appetite suppression, and altered taste preferences, but no study has successfully separated environmental constraints from intrinsic changes.

On Earth, LM relies on evidence-based, non-pharmacological interventions, particularly tailored nutritional strategies, to prevent physiological decline and optimize performance. Combined with precision medicine, which adapts care to genetic and metabolic profiles, this approach supports long-term health and can be directly applied to spaceflight challenges (Cahill and Hardiman, 2020). In the context of microgravity, framing astronaut nutrition as a form of LM means viewing preflight, in‐flight, and post‐flight dietary measures as a continuum of care. There currently appear to be no specific dietary guidelines or restrictions in place for astronauts during the pre- and post-flight phases (Morrison et al., 2021). Utilizing food and nutrition to mitigate harmful physiological changes associated with microgravity offers a promising alternative that can help limit the potential side effects of pharmaceutical interventions. In this context, a multiphase, conceptual framework, spanning pre-flight, in-flight, and post-flight stages, is outlined to illustrate how LM-based nutritional strategies could be structured across mission phases. While nutritional measures are already implemented, they have not yet been organized within an LM framework, highlighting an opportunity for future research to enhance astronaut health and recovery across mission phases.

Before launch, food systems undergo rigorous sensory and nutritional evaluation. Sensory analysis assesses appearance, flavor, texture, and aroma to ensure initial acceptability, and a diverse menu is formulated to prevent menu fatigue (Cooper et al., 2011). Yet, acceptability alone is not sufficient for long missions: preserving the nutritional integrity of spaceflight foods is critical, since time‐dependent nutrient degradation can compromise both in‐flight countermeasures and pharmacological adjuncts (Zwart et al., 2009).

In-flight nutritional countermeasures or during simulated microgravity have produced mixed results. However, increasing dietary protein intake and supplementing with amino acids, β-hydroxy-β-methylbutyrate, or antioxidant cofactors generally show promise in mitigating microgravity-induced muscle loss and strength decline (Gao and Chilibeck, 2020). Muscle atrophy in microgravity is primarily driven by disuse, which reduces mechanical loading and disrupts key mechanotransduction pathways. This leads to a shift in muscle protein turnover, characterized by increased protein degradation and decreased protein synthesis (Fitts et al., 2000). While promising evidence supports the use of targeted nutritional strategies, further research is needed to determine the optimal intake of protein and amino acids that can preserve muscle mass and function during spaceflight, without adversely affecting bone health. In particular, whey protein supplementation may help counteract spaceflight-induced metabolic disturbances such as hyperinsulinemia (Pal et al., 2010), hyperlipidemia (Bortolotti et al., 2011), and liver ectopic adipocytes (Bortolotti et al., 2011; Hamad et al., 2011). However, high protein intake must be carefully managed, as it may promote systemic acidosis and exacerbate bone demineralization, an already well-documented effect of spaceflight, unless buffered by appropriate alkali sources such as alkaline salts (Heer et al., 2017; Zwart et al., 2005). Moreover, in a 60-day study involving healthy men, participants were divided into two groups: one received a daily antioxidant and anti-inflammatory supplement, while the other received a placebo (Arc-Chagnaud et al., 2020). The supplementation failed to prevent losses in muscle mass and strength, highlighting its limited effectiveness as a standalone strategy. These findings call into question the adequacy of nutrition-based interventions alone for counteracting muscle deconditioning and underscore the importance of integrating them with complementary measures, particularly targeted exercise protocols. Spaceflight also induces metabolic disturbances, including glucose intolerance, insulin resistance, and increased cardiovascular risk. A key element in managing these issues is metabolic flexibility, defined as the body’s ability to switch efficiently between burning carbohydrates and fats to maintain energy balance, particularly during feeding-to-fasting transitions or when physical activity levels change (Palmer and Clegg, 2022). Microgravity and its associated inactivity can disrupt this adaptability, resulting in metabolic inflexibility, which exacerbates metabolic imbalances and undermines overall health in astronauts. Le Roux et al. (2024) reported that long-term spaceflight increases fasting carbohydrate oxidation while reducing lipid oxidation. These shifts from preflight values are likely driven by dietary modifications aboard the ISS and are linked to changes in body composition and inflight aerobic exercise. Despite these findings, there remains a significant gap in research on nutritional strategies to mitigate such metabolic disturbances during space missions (Gao and Chilibeck, 2020; Pittia et al., 2023).

How to proceed in practice? Although the development of closed loop bioregenerative systems and post‐flight rehabilitation protocols extends beyond conventional nutritional guidelines, a clear roadmap can still guide interdisciplinary teams. Bioregenerative life-support systems are being developed with the goal of closing essential habitat loops, including food production, CO_2_ reduction, O_2_ generation, waste recycling, and water management, thereby creating sustainable ecosystems for long-duration space missions (Häuplik-Meusburger et al., 2011; Zabel et al., 2016). Despite advances in this field, no current system has yet reached the level of maturity necessary to significantly enhance the autonomy of even a small lunar or Martian base (Johnson et al., 2021; Verseux et al., 2022). This underscores the challenge of scaling food production systems to reliably meet the crew’s dietary and energy needs during space missions. Beyond their fundamental role in nutrition and life support, onboard plant cultivation may also enhance psychological wellbeing and crew performance (Häuplik-Meusburger et al., 2011). Additionally, plants hold promising potential for biomanufacturing pharmaceuticals in space (McNulty et al., 2021). A long-term goal for future exploration missions is the potential for space-grown plants to contribute to a pharmaceutical life support system, enabling the on-demand production of high-value medical compounds that are difficult or impossible to supply through Earth-dependent systems. Moreover, given the physiological similarities between the effects of microgravity and those seen in aging populations (Mulavara et al., 2018; Strollo et al., 2018; Vernikos and Schneider, 2010), post-flight nutritional strategies may benefit from approaches used in rehabilitation and geriatric care. Since physical activity and exercise play a critical role in post-mission recovery, nutritional interventions should be integrated with these countermeasures to enhance overall effectiveness (Backx et al., 2017; Backx et al., 2018; Wu et al., 2021).

### 4.2 Physical activity and exercise

Physical activity encompasses all skeletal muscle movements requiring energy expenditure, and exercise is a structured and repetitive subtype of physical activity aimed at improving or maintaining fitness (e.g., running, swimming) (Caspersen et al., 1985). Physical activity also includes daily tasks, exercise, work, and leisure activities that involve thermogenesis, the energy produced from movements such as climbing the stairs or doing chores (Levine, 2002). The World Health Organization suggests that adults engage in at least 150 min of moderate-intensity aerobic activity (e.g., brisk walking, water aerobics) or 75 min of high-intensity physical activity (e.g., uphill walking, running) per week, as well as two sessions of muscle-strengthening activities per week (Bull et al., 2020; Singh et al., 2020). Taking part in physical activity for the purpose of conditioning or sports has been associated with a large array of reductions in health-related issues (e.g., decreased rate of morbidity and mortality in cardiovascular diseases, various cancers, improvement in brain health, and reduced obesity among many others benefits) (Piercy et al., 2018). However, these Earth-based exercise duration and frequency targets (and eventual benefits) may not be applicable to astronauts in microgravity (Moosavi et al., 2021). Indeed, astronauts, often compared to professional athletes (Hackney et al., 2015), usually train about 2 h daily, though requirements vary across space agencies (Lambrecht et al., 2017).

Microgravity in space poses unique challenges to maintaining muscle and bone health (Sibonga et al., 2015). The effects of microgravity exposure on the human body are numerous (Moosavi et al., 2021; Trappe et al., 2009). For example, research shows that compared to pre-flight, astronauts who come back from a mission present reduced muscle strength and volume (Bailey et al., 2018; Oganov et al., 1991; Puglia et al., 2018), increased joint stiffness (Lambertz et al., 2001), bone decalcification (with weight-bearing bones most affected (Moosavi et al., 2021; Nguyen et al., 2021; Smith et al., 2005)), and cardiovascular deconditioning (Coupé et al., 2009). Importantly, these health issues are not equally experienced by every astronaut (Fitts et al., 2010) and would not be limited to long-duration flights, with some muscle atrophy observed after only 9-day flights (Akima et al., 2000). These unfavorable effects on strength and endurance may affect an astronaut’s capacity to carry out duties both on a mission and upon return to Earth (Moosavi et al., 2021). In addition, prolonged head-down tilt bed rest, which involves a marked reduction in physical activity, has been associated with tiredness, sleepiness, insomnia complaints, monotony, and slower cognitive processing, and changes in emotional measures in healthy volunteers (Jiang et al., 2022). These findings suggest that reduced physical activity may interact with other LM pillars, such as sleep, cognitive performance, and stress resilience, highlighting a key research priority to better understand these interconnections in microgravity and analog environments. Unfortunately, many gravitational and time constraints may prevent astronauts from being able to train as much and as efficiently as they need to. For example, they sometimes must perform extravehicular activities or attend docking or undocking events, limiting their access to training devices.

According to Hackney et al. (2015), pre-mission training offers the greatest potential to maximize fitness and mission readiness. Individually tailored programs are therefore essential and should be adapted to duties and available time. In addition, pre-flight training is also an opportunity for health professionals (e.g., physiotherapists, sports scientists) to educate astronauts about the eventual in-flight training and the health issues they may face when in space. Astronauts learn to use in-flight devices effectively through guidance on posture, duration, and intensity (Lambrecht et al., 2017; Petersen et al., 2016). Preflight training generally focuses on building muscle mass and strength, as well as on increasing aerobic capacity to a level that is higher than that would be expected of someone their age. Guidelines aimed at improving muscle mass and strength as well as bone mineral density, three parameters that are negatively impacted by microgravity, often call for high-load resistance training. Nevertheless, time for physical training can at times be scarce during mission preparation and can involve travel, meaning that astronauts cannot always have direct access to exercise professionals or certain training equipment (Loehr et al., 2015).

Interestingly, based on a recent meta-analysis by Carvalho et al. (2022), muscle mass gains appear to be just as effective using low load as high load resistance training. Additionally, although in use for now several decades, especially in Japan, the last decade has seen a surge of research and application of a particular sub-set of low load training: low load blood flow restriction (BFR) training. BFR training, a technique combining low intensity exercise with BFR, has been shown to be safe and effective in eliciting gains in muscle mass and strength across multiple populations. Moreover, low load BFR resistance training has been shown to elicit bone formation and produce both aerobic and anaerobic improvements (Chua et al., 2022; Wang X. et al., 2023). It is important to note that the benefits of BFR do not necessarily go beyond those from high-load training. For example, a recent meta-analysis suggests that low load BFR training can be just as effective in eliciting muscle hypertrophy as high load training but may be even more effective in trained individuals (Geng et al., 2024). Another study revealed that while BFR enhanced squat strength and endurance in Australian soccer players, it did not yield superior performance in comparison to conventional training methods (Scott et al., 2017). However, in the absence of suitable training facilities, BFR training might be an effective way of enhancing astronauts’ muscle mass and strength while they are preparing for their mission. Due to travel and time constraints during mission preparation, training periodization, prescription and monitoring are often suboptimal, making remote tools for these tasks essential. The recent acceleration in growth of online training and telerehabilitation platforms has shown that telehealth can be an effective and efficient way to still provide quality training (Suso-Martí et al., 2021). Moreover, such a tele-training platform would allow astronauts to have access to their physical activity regimen regardless of their location and report on their training so that the exercise professionals can monitor, adjust and progress the program remotely.

While in space, astronauts need to exercise regularly to counteract the effects of prolonged microgravity exposure. Specialized exercise equipment is used on the ISS to help with this. Onboard the ISS, in-flight exercise devices currently include the Advanced Resistive Exercise Device (ARED), the T2 treadmill, and cycle ergometer with vibration isolation and stabilization system (CEVIS) (NASA, 2024b). Typically, astronauts perform more cardiovascular training in the early moments of a mission, and transition gradually to more resistance-based training. Astronauts generally increase their in-flight absolute workload for resistance exercise and treadmill running throughout their long-duration missions (Petersen et al., 2016). It is noteworthy that exercise countermeasures during space missions help reduce muscle loss and other health harms but cannot prevent them completely (Moosavi et al., 2021).

After their return from space, astronauts must readapt to Earth’s gravity, especially after longer missions. Postflight reconditioning is in fact implemented from day one of landing and for the following 21 days (depending on spatial agencies). When they land, astronauts exhibit (among other things) significant deficits in manual dexterity, dual-tasking, motion perception, vehicle operation ability, and often lower back pain (Lambrecht et al., 2017; Moore et al., 2019). The postflight training aims to restore any residual performance loss and facilitate readaptation. It teaches them to move again normally, to increase their reaction speed and to control fast movements that are not necessary in microgravity (see (Lambrecht et al., 2017; Petersen et al., 2017) for a detailed account of the post-flight training program). Although exercise is beneficial in this process, high load exercise may be contraindicated and even detrimental as the tissues cannot be heavily loaded due to deconditioning. Implementation of BFR training in this phase would allow for reduced loading of the tissues while providing an effective stimulus for muscle mass and strength reconditioning. Moreover, after an initial period, astronauts will be returning to their home location to continue the reconditioning phase. A tele-training platform could allow for close monitoring of the training performed and enable the exercise professionals to continuously adjust and progress training in the most appropriate manner through a remote feedback mechanism.

Future missions involving small spacecraft, such as Orion, will not be able to accommodate large devices like ARED, meaning that current musculoskeletal countermeasures will become partly obsolete. A promising alternative is the compact flywheel device, which offers strength and power benefits (Canadian Space Agency, 2023). Due to their efficiency at low loads, BFR methods combined with tele-training may provide practical countermeasures, particularly given that signal delays increase with distance from Earth.

### 4.3 Sleep

Sleep is a fundamental physiological process critical for maintaining physical and mental health, influencing both cognitive function and physical performance (Tahmasian et al., 2020; Charest and Grandner, 2020). It is regulated by the interaction of homeostatic sleep pressure and circadian rhythms (Borbély, 1982). The National Sleep Foundation recommends 7–9 h of nightly sleep for adults to maintain optimal functioning (Hirshkowitz et al., 2015). Circadian rhythm, primarily regulated by the suprachiasmatic nucleus of the hypothalamus, follows an endogenous ∼24-h cycle that synchronizes physiological processes, including the sleep-wake cycle, with environmental cues such as light (Dijk and Czeisler, 1994). While optimal cognitive and physical performance is desirable for most individuals, it is essential for astronauts who must execute highly precise and demanding tasks during space missions under extreme and often unpredictable conditions. Pre-flight strategies therefore focus on circadian alignment and sleep optimization through individualized sleep assessments, controlled light exposure, and scheduling adjustments to strengthen circadian robustness before launch. These preventive measures are critical to reduce vulnerability to circadian misalignment once in orbit.

Exposure to the unique conditions of spaceflight including microgravity, altered light-dark cycles, and isolation can significantly disrupt circadian rhythms and sleep regulation (Flynn-Evans EE. et al., 2016; Guo et al., 2014; Zong et al., 2025). These disturbances may impair physiological stability and operational performance, underscoring the need for targeted countermeasures to optimize health and functionality during extended missions in space (Guo et al., 2014). The circadian clock orchestrates the temporal organization of a wide range of physiological and behavioral processes, including brain waves patterns, sleep-wake regulation, thermoregulation, endocrine rhythms, cardiovascular dynamics, cellular turnover, metabolic pathways, and behavioral patterns, all of which exhibit approximately 24-h periodicity (Bell-Pedersen et al., 2005). Both sleep quality and quantity are crucial for physiological system function, impacting cardiovascular, mental, cognitive, memory, immune, reproductive, and hormonal regulation (Charest and Grandner, 2020; Lloyd-Jones et al., 2022). Sleep emerges from the interaction between voluntary behaviors, such as light and noise reduction, and involuntary physiological processes, including melatonin secretion and neural activity shifts (Kryger et al., 2021). Disruption of either domain can compromise sleep quality (Grandner, 2022).

Astronauts face unique stressors in space, requiring adaptation of sleep, health, and performance guidelines for their unique environment, unlike Earth-specific guidelines. Circadian regulation becomes compromised in space or microgravity conditions due to changes in environmental cues and downstream effects on clock gene expression, which impairs internal timekeeping mechanisms (Ferraro et al., 1989; Hoban-Higgins et al., 2003; Monk et al., 1998). For example, the ambient lighting intensity aboard the space station is substantially lower than the recommended terrestrial levels (2,500 lux), which can affect their circadian rhythm (NASA, 2023a). Therefore, in the absence of Earth-like environmental light-dark time cues, astronauts experience circadian disruption and behavioral desynchrony (McPhee et al., 2009; Piltch et al., 2025). Furthermore, the unique electromagnetic environment and heightened radiation exposure encountered in space, compared to Earth, may contribute to perturbations in the regulation of circadian rhythms (Martel et al., 2023). Circadian misalignment and sleep disruption affect multiple physiological systems, including neural, musculoskeletal, endocrine, and cardiovascular functions (Sletten et al., 2020), and in astronauts, these effects increase health risks and impair cognitive and psychomotor performance, compromising mission-critical tasks (Guo et al., 2014). Disrupted sleep and circadian misalignment may also intensify stress reactivity, impair mood regulation, and reduce motivation for physical activity (Sleep, 2025), illustrating how sleep interacts with other LM pillars to sustain astronaut health.

Evidence from both simulated and real spaceflight shows that circadian rhythms can be disrupted, likely due to altered environmental cues and changes in internal regulatory mechanisms (Ferraro et al., 1989; Monk et al., 1998; Sulzman et al., 1992; Basner et al., 2013). Attenuation of the circadian rhythm in core body temperature has been documented during space missions, supporting the hypothesis that microgravity environments interfere with normal thermoregulatory and circadian processes (Basner et al., 2013). Bed-rest protocols, commonly used to simulate the effects of microgravity on cardiovascular and systemic physiology, have demonstrated alterations in circadian rhythmicity across multiple physiological parameters. Findings from these studies indicate that simulated weightlessness disrupts normal circadian regulation, likely through changes in autonomic balance, endocrine signaling, and reduced external time cues (Ferraro et al., 1989; Mizuno et al., 2005; Shiraishi et al., 2003). In parallel, bed rest has been shown to alter the circadian secretion patterns of key hormones and electrolytes, including cortisol, melatonin, and aldosterone (Liang et al., 2012). Even minor shifts in the circadian phase can substantially impact human performance (Burgess et al., 2013). In contrast to conditions on Earth, the space environment presents a unique combination of altered gravity, altered light exposure, and other stressors that interact in complex ways. A long-term study conducted on astronauts aboard a space station demonstrated a significant reduction in the amplitude of circadian rhythms for both oral temperature and alertness, indicating a dampening of physiological rhythmicity during extended spaceflight (Monk et al., 2001). Dijk et al. (2001) investigated mood and cognitive performance changes in five astronauts before, during, and after space missions lasting 10–16 days, during which average daily sleep duration was approximately 6.5 h. The study found a decline in cognitive performance and mood during missions, possibly due to disruptions in circadian regulation caused by the absence or alteration of gravitational forces compared to Earth’s constant gravity (Dijk et al., 2001). Although it remains challenging to isolate the effect of individual variables in orbit, findings from Earth-based simulation studies suggest that the absence or alteration of gravitational forces compared to Earth’s constant gravity may significantly contribute to disruptions in circadian regulation.

Beyond circadian rhythm disruption, sleep disturbance represents a significant physiological challenge for astronauts in orbit. Data from surveys of space shuttle crew members indicate that the most pronounced sleep disruptions typically occur during the first and final days of the mission. On average, astronauts obtain less than 6 h of sleep per 24-h period, falling short of the recommended duration for optimal performance and recovery (Santy et al., 1988). In space, astronauts typically experience prolonged sleep onset latency compared to conditions on Earth (Putcha et al., 1999). For example, a wide range of sleep disturbances have been documented during Skylab, Space Shuttle, and Mir missions, including reduced sleep duration, altered circadian rhythms, and increased sleep fragmentation (Barger et al., 2014). In addition to the reduced total sleep duration, alterations in sleep architecture are commonly observed during short-term space missions. These changes include a decrease in slow-wave sleep and rapid eye movement (REM) sleep, a shortening of REM sleep latency, and an increased frequency of nocturnal arousals (Dijk et al., 2001). In contrast, long-duration space missions, both sleep onset latency and the latency to deep sleep stages are significantly prolonged. Following return to Earth, sleep latency and REM latency are markedly reduced, while the proportion of REM sleep is substantially increased, particularly during the first polysomnographic recording post-landing (Dijk et al., 2001).

An analysis of medication usage across 79 Space Shuttle missions revealed that 45% of administered drugs were intended for the management of sleep disturbances (Putcha et al., 1999). Over 70% of astronauts on Space Shuttle and ISS missions use pharmacological sleep aids as a countermeasure to manage sleep disturbances during spaceflight (Barger et al., 2014). While these agents facilitated sleep onset, they did not significantly extend total sleep duration. Findings indicated that melatonin significantly reduced sleep latency compared to placebo; however, no significant differences were observed in other sleep architecture parameters. Commonly used sleep medications during space missions include zolpidem, zaleplon, extended-release zolpidem, and flurazepam, while less frequently used medications include temazepam, eszopiclone, melatonin, and quetiapine fumarate (Barger et al., 2014; Whitmire et al., 2013; Jing et al., 2014). Stimulants may be administered during spaceflight to counteract excessive sleepiness and maintain wakefulness during critical operational tasks. Multiple pharmacological interventions are available to support the management of sleep disorders (Morin et al., 2024). Dual Orexin Receptor Antagonists (DORAs) constitute the most recent class of pharmacological agents developed for the management of sleep disorders, acting by selectively inhibiting the activity of orexin neuropeptides involved in the regulation of wakefulness (Herring et al., 2016; Mignot et al., 2022; Yardley et al., 2021). While their use has not yet been investigated within the context of space missions, DORAs represent a promising area for future research aimed at promoting optimal sleep, which remains a fundamental pillar of lifestyle-based therapeutic strategies (Mogavero et al., 2023).

Space agencies implemented countermeasures to help astronauts prepare for their missions. As part of this preparation, astronauts with altered wake times on launch day were exposed to a light-dark regimen to minimize sleep loss. This allows their circadian rhythms to shift to the required phase (Whitson et al., 1995; Czeisler et al., 1991). To mitigate the adverse effects of circadian misalignment and sleep deficiency during flight, various countermeasures and therapeutic interventions are implemented to preserve health and operational performance (Chabal et al., 2025). Light exposure is a widely utilized intervention for managing circadian misalignment and sleep disturbances. To facilitate circadian training, protocols often incorporate the use of bright light to enhance, and dark goggles to minimize, photic input to the circadian system. Short-wavelength light, particularly in the blue to green spectrum (∼460–512 nm), has been shown to be more effective than broad-spectrum bright light in modulating the phase and amplitude of the human circadian rhythm (Barger et al., 2012). Due to the impact of intensive workloads and irregular, shift-based work-rest cycles on circadian regulation and sleep quality, the implementation of strategically optimized schedules has proven effective in mitigating fatigue and enhancing both vigilance and operational performance in astronauts (Williamson et al., 2011). Thus, non-pharmacological strategies such as light therapy to regulate circadian rhythms, and optimization of work-rest schedules to support adequate sleep duration and quality (Wu et al., 2018) should be prioritized. Pharmacological agents to induce sleep or maintain wakefulness, may be used, when necessary, but typically serve as adjuncts rather than primary strategies.

Historical records from the Apollo 7 mission document a case in which an astronaut, experiencing severely disrupted sleep, fell asleep while on duty and subsequently required 5 mg of amphetamine to remain alert (National Aeronautics and Space Administration, 1968). In post-flight interviews, Whitmire et al. (2013) reported that 75% of astronauts had used stimulants such as caffeine or modafinil at some point during their missions to sustain alertness and cognitive performance. Ground-based studies have demonstrated that caffeine effectively mitigates declines in alertness, cognitive function, and operational performance associated with sleep deprivation. Its benefits are particularly evident in emergency scenarios requiring prolonged wakefulness or rapid transitions from sleep to wakefulness (Wyatt et al., 2004). In comparative studies of stimulant efficacy under conditions of sleep deprivation, Killgore et al. (2008) and Killgore et al. (2009) found that caffeine, modafinil, and dextroamphetamine all significantly enhanced performance relative to placebo. Caffeine produced the most rapid onset of performance improvement but was associated with the greatest incidence of adverse effects. Dextroamphetamine exhibited the longest latency to efficacy and was linked to disrupted recovery sleep. Modafinil, in contrast, demonstrated performance benefits without notable side effects (Estrada et al., 2012).

Although multiple countermeasures have been investigated, the literature does not indicate a clear consensus on which approach is the most effective. Recovery protocols such as gradual re-exposure to natural light-dark cycles and continued monitoring of sleep quality may help support post-mission readaptation. While some evidence suggests that alterations in sleep architecture after spaceflight are transient (Piltch et al., 2025), systematic evaluation of structured recovery protocols remains limited and should be prioritized in future research. Further research is required to evaluate the efficacy, safety profile, and optimal administration protocols of sleep medications during orbital missions. This will facilitate the development and selection of individualized pharmacological interventions and stimulants with improved safety and effectiveness for astronaut use. In-flight studies assessing the effects of circadian disruption and sleep deficits on performance remain limited. Therefore, systematic investigations are needed to elucidate the relationship between circadian and sleep disturbances and operational efficiency. Future exploration missions, particularly to destinations other than Mars which has a day-night cycle (24 h 39 min) analogous to Earth’s will face significant challenges in modulating circadian clock robustness to accommodate novel light-dark environments. Research on sleep disturbances in aerospace medicine is crucial for astronaut safety, health, and performance, and offers insights applicable to terrestrial populations.

### 4.4 Stress

Psychological stress can be defined as the response of an organism to an identified demanding stimulus or a threat to homeostasis, that is the internal conditions aimed to be maintained by the body (McEwen and Stellar, 1993; Goldstein and Kopin, 2007; Kolbell et al., 1995). Unlike other health-related factors such as sleep, exercise, or nutritional intake, there is no specific recommended amount of stress that individuals should limit themselves to. In fact, stress is experienced by everyone daily and is an inherent part of human experience (Hutmacher, 2021; Fink, 2010). Typically, stressful situations may activate two main components, namely, a release of corticotropin hormone (CRH) and activation of the locus coeruleus-norepinephrine autonomous system (Chrousos, 2000). In turn, both systems activate a diversity of responses that may lead to behavioral and peripheral changes that improve an organism’s ability to adjust homeostasis and increase chances of survival. This cascade of effects is observed through the hypothalamic-pituitary-adrenal axis, which enables release of glucocorticoids to activate short-term physiological responses to stress. However, intense, prolonged stress can induce significant problems. High glucocorticoid levels have in fact been related to the atrophy of certain brain regions, but also to a reduction in the feedback response, via the hippocampus, on CRH secretion (Frodl and O’Keane, 2013). These changes can be either functional or structural (McEwen, 1999) and can cause cognitive disorders (Li et al., 2008) if they rise above a given threshold, which varies amongst individuals (Sandi, 2013). On the long term, this can also result in further excessive cortisol secretion and induce a cascade of hippocampal damage (i.e., the “glucocorticoid cascade hypothesis” (Sapolsky et al., 1986)). Such can lead to health problems, including chronic disease and tissue damage (Selye, 1978). In addition, stress can lead to performance problems, such as difficulty concentrating (LeBlanc, 2009; Chu et al., 2025), memory problems (Gould and Tanapat, 1999), chronic fatigue (Chu et al., 2025), and a poor ability to cope with future stressful events (McEwen, 2007). In that regard, the ACLM underscores the necessity of integrating stress-reduction techniques (American College of Lifestyle Medicine, 2024). However, the stress response amongst individuals varies vastly (Selye, 1975; Yaribeygi et al., 2017; Schneiderman et al., 2005). Stress is indeed a highly complex phenomenon. Accordingly, effective stress management necessitates an understanding of the reciprocal nature of brain-body communication in addition to neuroendocrine mechanisms. The gut-brain axis shows how gut microbiota has the potential to influence stress responses (Xiong et al., 2023), connecting diet and mental health in ways that are especially pertinent for astronauts in confined spaces.

Space missions are characterized by many stressful situations including but not limited to microgravity, isolation, confinement, noise, and circadian rhythm disturbances (Oluwafemi et al., 2021; Kanas and Manzey, 2008), and these stressors can manifest across multiple domains of human functioning. The complex nature of the tasks carried out by astronauts further induces stress given the criticality of these tasks for the success of the mission and, to some extent, for their colleagues and their own survival. Different work management styles among astronauts can also lead to misunderstandings, communication issues, and conflicts (Oluwafemi et al., 2021; Kraft et al., 2003), and the rejection ensuing from this may also lead to stress (Del Giudice et al., 2011). Taken together and considering that stressors may have a composite/additional effect with each other (Gatti et al., 2022), these factors can exert deleterious impacts on mental and physical health. NASA has developed the Lifetime Surveillance of Astronaut Health (LSAH) project, which captures information from flight surgeon or crew surgeon notes taken during weekly private medical conferences (NASA, 2023b). Thanks to this data and to statistical projections made possible through the Integrated Medical Model (IMM), NASA can extrapolate and infer prevalence of mental and psychological health issues. Data from the LSAH shows that symptoms of anxiety and depression occurred during space flight, approximately once every 1.2 years for symptoms of anxiety and once every 7.2 years for symptoms of depression (NASA, 2025a). Extrapolation computed for the IMM also indicates an 85.2% chance of a female astronauts meeting Diagnostic and Statistical Manual of Mental Disorders criteria for anxiety and a 22.8% chance for male astronauts (Evidence Report, 2016).

Literature on mental health proposes many different strategies to mitigate the negative impacts of stress over mental and physical health. Generally, having a positive outlook on life can help improve the overall health of one’s wellbeing and therefore can improve stress management (McEwen, 2006). Behavioral strategies such as maintaining a healthy diet, avoiding smoking, being active regularly (Rovio et al., 2005; Bernadet, 1995), and having positive social support can also lead to better stress management (McEwen, 2006). Stress management techniques have also been developed to help individuals to be able to better face stressful situations, both for their professional or personal lives. Work is an omnipresent component of space crews. Typically, an astronaut’s day lasts 16 h including between 6 and 10 h of scientific work, 2 h of physical activity and about 3 h for leisure and maintenance (National Aeronautics and Space Administration, 2006). A meta-analysis conducted by Richardson et al. focused on determining the effectiveness of stress management interventions in occupational settings (Richardson and Rothstein, 2008). They found a significant medium to large effect size of these interventions on stress outcomes, but with important moderating effects depending on the stress management technique. Cognitive-behavioral interventions (e.g., acceptance commitment therapies, stress inoculation training or rational-emotive therapy) systematically produced larger effect sizes than other types of techniques (e.g., relaxation, organizational, multimodal and alternative interventions). A more recent meta-analysis assessed the effects of stress management training for occupational settings focusing specifically on cognitive-behavioral skills training, relaxation techniques and combination of techniques (Kröll et al., 2017). They outlined that these techniques were positively related to psychological health and that they offered employees new abilities to cope with stressors in the workplace and, therefore, to perceive situations as being less stressful. They also found that larger effect sizes were observed for relaxation techniques. To identify the most effective stress management interventions for astronauts, it is essential to consider not only occupational stress management studies, but also those focused on everyday life. This is because astronauts live and work in the same environment, blurring the line between professional and personal stressors. Similar to occupational stress management techniques, personal stress mitigation strategies also include meditation (Tang et al., 2009), cognitive-behavioral-based methods (Nakao et al., 2021), listening to music (Khalfa et al., 2003), and even biofeedback approaches (Kudo et al., 2014). For example, Gaab et al. (2003) showed that cognitive-behavioral stress management training was efficient for reducing both endocrine and psychological responses to an acute stress intervention. Different meta-analyses were conducted for some of these interventions, raising conflicting results as to the clinical impacts on acute stress response (Goyal et al., 2014; Chiesa and Serretti, 2009). Recently, Rogerson et al. (2024) examined the impact of mind body therapies, mindfulness methods, relaxation therapies, and talking therapies (including cognitive-behavioral interventions) on cortisol secretion levels, used as a proxy for acute stress. They found that, overall, these interventions outperformed pooled active (as opposed to passive) control conditions with a medium positive effect size. The largest effects were observed for mindfulness, meditation, and relaxation interventions. In contrast, mind body practices and talking therapies failed to exert a significant effect. These findings should however be taken with caution given their sole focus on cortisol-based measures rather than incorporating the subjective component of the stress response.

Considering these interventions applicable on Earth, questions arise as to what action may be taken to support and reduce stress among astronauts. For example, mindfulness and relaxation practices have emerged as key tools for managing stress in space for long-duration missions (Pagnini, 2024). These practices may help astronauts stay present, focus on their missions, bolster mental resilience, and manage stress, which is particularly beneficial in an environment where stressors are constant and inevitable. Yin et al. (2023) discussed different strategies raised by Kanas (2015) and other sources that can be applied to ensure better coping abilities among astronauts for pre-flight preparation, in-flight support, and post-flight readaptation. As a pre-flight preparation key step, they outlined how candidate selection should consider various factors related to stress management including personality, intercultural and interpersonal skills, and performance on tests, simulations and interviews in which stress management can be assessed. Astronauts should also undergo psychological and emotional training as it has been previously related to reduced negative emotional stress in highly uncertain situations (Hodzic et al., 2015).

In-flight countermeasures typically include monitoring and supporting the wellbeing of astronauts. Astronauts can be encouraged to reflect upon their own stress level. External monitoring is also frequent. Due to the documented reluctance of astronauts toward being monitored on their emotions, combinations of approaches may be useful. For example, stress reactions can be assessed using a set of behavioral, subjective self-reported and even physiological measures less prone to subjectivity or to one’s control including methods such as facial analysis, eye tracking or other low-invasive portable devices (e.g., smartwatches, garments or bands that can collect electrocardiography, respiration or other physiological responses related to stress (Frazier and Parker, 2019; Dorsey et al., 2022; Crosswell and Lockwood, 2020)). Personal leisure-time activities should also be offered as they provide psychological detachment from work, which in turn helps facilitate stress recovery (Sonnentag and Fritz, 2007). Given that homesickness and distance from loved ones may represent an important stress factor, quality of life and habitat design should also be promoted. Strategies for this may include, for instance, carrying personal items onboard and working/living in an ergonomic and comfortable environment (Burattini et al., 2014). Better supporting astronauts’ family as well as ensuring proper communications with Earth, for family but also for psychological support, is also key to improving stress management (Kanas et al., 2009; Manzey, 2004). Post-mission readaptation finally involves caring for astronauts once they have returned to Earth. Mediatic attention, family reuniting and other integration difficulties may arise, but structured counseling, debriefings and adequate time for recovery may help facing these challenges (Kanas, 2015).

Some components for stress management may finally be specific to long-duration travel (Gatti et al., 2022). The Controlled Ecological Life Support System (CELS) study (Yuan et al., 2019) and the Mars500 project (Basner et al., 2014) provide key insight on these aspects. They involved isolating four volunteers for a 180-day period for a Mars-like day-night simulation (CELS study) or isolating six volunteers for a 520-day simulated trip to Mars (Mars500 project). In both studies, no increase in stress levels or negative emotions were found, suggesting emotion adaptation as an internal defensive system toward adversity (Wang et al., 2014). Isolated and confined environments are, however, highly known for the stress they may induce, and regardless of human adaptation, stress may still be experienced. In that regard, specific countermeasures should still be integrated in long-duration space travel. Multiple aspects must, however, be considered. Due to important communication delays between Earth and Mars, the crew needs to be autonomous and able to resolve conflicts, if they are to arise in the group (Oluwafemi et al., 2018; Bell et al., 2019). Conflict resolution methods vary across cultures, especially in confined spaces. Mission research should focus on identifying effective coping strategies, resources, and self-regulation techniques for optimal performance (Dinges, 2025). These aspects are of uttermost importance due to specific effects known to arise on the long term, including for instance the “disappearing Earth phenomenon” which represents the growing and progressive homesickness and melancholy associated with increasing distance from Earth.

### 4.5 Social connection

Social connection refers to the extent to which an individual is connected to others. Social connection can be conceptualized as a continuum, where higher level represents a protective health factor and lower-level increased risk (Holt-Lunstad, 2018). The construct of social connection encompasses three interrelated but distinct dimensions: structural (e.g., network size, roles, marital status, living arrangements, frequency of contact), functional (e.g., the provision and receipt of social support), and qualitative aspects (e.g., relationship satisfaction, intensity, and reciprocity) (Holt-Lunstad, 2018). Even if these three components are linked to health, they are not highly correlated, meaning that an individual may have a large social network (structural aspect), but receive little support (function aspect) or experience low satisfaction from those relationships (Holt-Lunstad, 2018). As such, social connections can act both as a protective factor and a risk factor for health, as they may also be a source of interpersonal tensions, conflict, stress, low cohesion, or inadequate social support. Literature provides extensive evidence that social connection is a key determinant of psychological, cognitive, and physical health. Indeed, isolation and loneliness have been linked to an increase occurrence of multiple psychopathologies, such as depression, stress disorder, burnout, anxiety, and post-traumatic stress disorder (Holt‐Lunstad, 2024). Moreover, having satisfactory social connections is associated with lower risks of developing dementia. Social support can positively influence physical health while unsatisfactory social connections increase the risks of heart disease, stroke, hypertension, and even mortality (Holt‐Lunstad, 2024). Isolation and loneliness are associated with 32% and 15%, respectively, increased chances of earlier death (Wang F. et al., 2023). Similarly, Holt-Lunstad et al. (2010) found that being socially connected increases survival rates by 50% and demonstrated that social connections are an important predictor of mortality, with an effect size comparable to other major risk factors such as obesity, smoking and physical inactivity. Recently, Holt‐Lunstad (2024) outlined three mechanisms through which social connections influence health outcomes, including illness and mortality. First, the psychological pathway suggests that social connections serve as a psychological buffer, reducing the negative effects of stress, enhancing resilience, and fostering a sense of purpose and safety. Second, the behavioral pathway indicates that individuals who are more socially connected are more likely to engage in health-promoting behaviors, such as regular physical activity, balanced nutrition, sufficient sleep, and adherence to medical treatments. Third, the biological pathway emphasizes the role of physiological processes, highlighting how strong social ties are associated with better cardiovascular, neuroendocrine, and immune system functioning (Uchino, 2006).

Social connections have been extensively studied and promoted as a pillar of health on Earth (Martino et al., 2015). However, extreme isolation and confinement inherent to space exploration create unique social stressors, such as social monotony, crew tensions, and interpersonal conflicts (Le Roy et al., 2023). Additionally, factors such as crew composition, communication, and leadership also shape astronauts’ social connections (Bell et al., 2019). The extreme and confined living and working conditions of encapsulated environments foster social monotony and a lack of privacy, which can negatively affect team dynamics by increasing interpersonal tensions, reducing cohesion, and triggering conflict (Holt-Lunstad, 2018). Prolonged separation from family and friends impedes astronauts of access to key sources of social support that are typically available on Earth (Johnson et al., 2012). Quantitative and qualitative studies have shown that social stress tends to be the main stress dimension affected by isolation and confinement (Wang F. et al., 2023). Furthermore, reduced cohesion may contribute to sleep disturbances and lower engagement in health-promoting behaviors (Barbour et al., 2025), illustrating interactions across lifestyle domains.

As mission durations have shifted from short-term flights during the shuttle era to long-duration stays aboard the ISS, and now towards even longer deep space missions to Mars, astronauts are increasingly exposed to the cumulative effects of psychosocial stressors. Mission length is hypothesized to be a key factor influencing social dynamics, as several indicators of crew functioning have been reported to decline over time (Kanas et al., 2009). This suggests that spaceflight impacts short-duration crews differently than long-duration crews. For instance, astronauts tend to spend less time with their crewmates during longer missions (>30 days) compared to shorter missions (Bell et al., 2019), potentially reflecting a decrease in social cohesion, which may contribute to increased perceptions of isolation. While some studies have reported an increase in crew tensions and conflict (Struster, 2010; Nicolas et al., 2016a; Golden et al., 2018), others have not found any specific trend over time (Bell et al., 2019). However, crews will inevitably experience conflict or interpersonal tensions at some point throughout the mission (Bell et al., 2019). As work overload has been reported (Flynn-Evans E. et al., 2016), it can increase the likelihood that task disagreements between crewmembers will lead to interpersonal conflict and emotional distress (Somaraju et al., 2022). Additionally, crews tend to experience a decrease in cohesion, particularly when subgroups develop, which can potentially threaten crewmembers’ wellbeing and performance (Nicolas et al., 2016b; Palinkas and Suedfeld, 2021). Palinkas et al. (2004) showed that social support from both crewmates and family declined over time, with help-seeking from colleagues sometimes worsening anxiety and depression due to shared stressors depleting the crew’s emotional resources. While social connections can promote wellbeing, they may also become a source of stress when unsatisfactory. Although team cohesion is known to enhance performance in high-intensity teams down on Earth, its impact in space remains unclear, suggesting that space crews may function differently (Bell et al., 2019). Therefore, any countermeasure to support positive social dynamics during spaceflight must be tailored to the unique context of space missions.

Astronaut selection and crew composition play a critical role in shaping interpersonal dynamics, fostering positive social connections, and enhancing group cohesion. The ability to work in a team is assessed during the selection process and has been identified as an important requirement for astronauts’ role (Landon et al., 2018). In a report prepared for NASA, Landon (Landon, 2022) identified key personality traits, such as emotional stability, conscientiousness, resilience, adaptability to diverse situations and cultures, motivation, and team orientation, as crucial for individuals to successfully live and work in space. While optimal team composition is important for ensuring compatibility and crew functionality, research on heterogeneity versus homogeneity in terms of culture, gender, age, background, and values remains inconclusive regarding which approach is most effective (Bell et al., 2015). The assignment of a commanding role is also critical, as leadership is essential for maintaining group harmony and providing hierarchical support (Palinkas and Suedfeld, 2021).

Pre-flight preparation, including field exercises, team-building activities, seminars, and extensive joint mission preparation, helps astronauts develop strong teamwork skills (Gatti et al., 2022). For instance, NASA’s field activities aim to develop “Expeditionary Field Skills,” which encompass leadership/followership, communication, self-care, team-care, teamwork, and small-group living skills (Landon et al., 2018). Numerous countermeasures have also been implemented during spaceflight to maintain communication with loved ones on Earth, aiming to provide social support and reduce feelings of isolation. These include weekly video conferences, regular phone calls and emails, delivery of personal care packages, and bi-weekly psychological support sessions (Sipes et al., 2019). While these countermeasure strategies are presumed to help maintain social connections, to our knowledge no research has been conducted to measure their effectiveness. Deep space missions, such as those to Mars, will introduce unprecedented psychosocial stressors. A round trip could last about 2 years, during which astronauts will, for the first time in humankind, see Earth as a distant star, potentially increasing feelings of isolation and disconnection (Gatti et al., 2022). This will also prevent them from experiencing the salutogenic psychological effects typically tied to Earth observation from the ISS (Ritsher et al., 2007). Communication delays will also make real-time communication with loved ones and mission control more difficult, requiring greater crew autonomy. This may foster group thinking and lead to project interpersonal tensions toward mission control as a coping mechanism (Gatti et al., 2022; Gushin et al., 2016). Unlike ISS missions, where crew rotations can create some social novelty and counteract the social monotony, no crew rotation is envisioned for deeper space missions. Enhancing crew autonomy is a promising approach to reduce conflicts with mission control and has been linked to improved mood, an essential factor as deep space exploration will inherently demand greater self-sufficiency (Palinkas and Suedfeld, 2021). Increasing autonomy may also promote a sense of control and environmental mastery, defined as the ability to influence or adapt one’s surroundings, which has been shown to support social adaptation in isolated environments (Nicolas et al., 2022). Technology-assisted countermeasures, including artificial intelligence (AI) based virtual assistants and virtual reality systems, offer alternatives to traditional psychosocial support. AI social robots can provide emotional support, active listening, and companionship, while virtual reality (VR) can recreate Earth-like environments to help astronauts maintain a psychological connection with home (Gatti et al., 2022). Though still in development, these tools show promising early results. Additionally, individual and group self-help interventions, like telehealth on Earth, may enable astronauts to monitor their own and their crewmates’ psychological health, while enhancing coping and self-regulation skills (Palinkas and Suedfeld, 2021).

Reintegration after space flight represents a critical moment as many challenges can be experienced for both astronauts and their families. Indeed, reunion after long term separation requires reshaping family dynamics and re-establishing interpersonal roles. Research on how absent astronauts experience these challenges is scarce (Johnson et al., 2012). However, studies have shown this period may involve reduced mood and performance, and in some cases, major depressive disorders, anxiety, or substance use requiring medical and psychological support (Le Roy et al., 2023). Therefore, providing sustained support to astronauts and their families during this reintegration phase is recommended to facilitate adaptation and sustain healthy social connections after long-duration missions.

### 4.6 Avoidance of risky substances

According to the World Health Organization, risky substance use includes the consumption of tobacco, alcohol, illicit drugs, and the inappropriate use of prescription medications in ways that impair physical health or social functioning (AFRO, 2025). The ACLM further highlights that such substances are major contributors to chronic diseases and premature mortality (American College of Lifestyle Medicine, 2024). On Earth, definitions of harmful alcohol consumption vary across countries, but there is increasing scientific consensus that no level of alcohol use is completely risk-free (WHO, 2025; HSS, 2025). In space, however, environments are highly controlled, and the use of alcohol is strictly limited or prohibited (STD NASA, 2022). By contrast, prescription medications play an essential role in maintaining crew health and operational performance (Jaworske and Myers, 2016) as previously discussed in the section focused on sleep. However, while many substances are prohibited, the extreme psychological stressors of long-duration and deep-space missions could increase the theoretical risk of misuse if any substances were available, underscoring the importance of prioritizing psychological rather than pharmacological coping strategies.

As missions extend beyond low Earth orbit, new challenges emerge. Isolation, disrupted circadian rhythms, and mission-related stress are known to affect mental wellbeing (Kanas, 1998; Flynn, 2005; Kalb and Solomon, 2007; Kandarpa et al., 2019; Marazziti et al., 2022). Programs such as the Artemis missions (NASA, 2025b) or future Mars expeditions introduce additional stressors, including prolonged communication delays and the lack of rapid emergency medical help. These conditions increase the need for strict medication stewardship and autonomous health management systems to protect astronaut safety during long-duration missions. The deterioration of other lifestyle pillars may also lead to increased reliance on pharmacological support, as these pillars are closely linked to mental and physical health. Their deterioration in spaceflight conditions, such as disrupted circadian rhythms, limited physical activity, isolation, and stress, can exacerbate psychological stress and increase the reliance on pharmacological treatments as well. This is particularly evident in the management of sleep disturbances, where the use of hypnotics has become common practice during missions (Barger et al., 2014). Nearly three-quarters of astronauts have reported using sleep medications during missions, primarily zolpidem (a non-benzodiazepine hypnotic), or temazepam (a benzodiazepine with sedative properties) (Putcha et al., 1999; Wotring, 2015). Although intended for short-term treatment of insomnia, both medications carry a high-risk of dependence (Petursson and Lader, 1981; Terzano et al., 2003), and potentially misuse, especially if used repeatedly over extended periods.

Beyond sleep-related issues, other psychological vulnerabilities associated with isolation and confinement have been well documented. Studies of other isolated and confined environments, such as Antarctic research stations and submarines, have identified common patterns of psychological adaptation among crews (Kanas, 1998; Rohrer, 1961). One model describes three distinct stages of reaction to prolonged isolation (Rohrer, 1961). The first stage is characterized by anxiety, which usually lasts a few weeks while individuals adapt to their new surroundings and social dynamics. The second stage is characterized by boredom, demotivation and depressive symptoms, which often emerge once the initial excitement has faded, and routines have become monotonous. Finally, as the mission nears its end, a third phase may occur where crew members experience euphoria and a heightened mood driven by the anticipation of returning home (Rohrer, 1961). These phases may be amplified in deep space missions, where confinement is prolonged, communication with Earth is delayed, and the environment becomes even more monotonous. Under such conditions, the risks of psychological strain are expected to increase, raising important questions: How will astronauts cope with emotional distress in the absence of real-time psychological support? Could the combination of isolation, stress, and limited coping mechanisms lead to an increased temptation for self-medication, particularly during multi-year missions such as those planned for Mars? Historical accounts, such as those shared publicly by Buzz Aldrin in his memoir, illustrate the stigma and long-term challenges of depression and alcohol use following space missions (Telegraph, 2025; Biography.com, 2020). While these experiences occurred post-mission rather than in-flight, they highlight the importance of destigmatizing mental healthcare and substance use disorders in astronaut population. Doing so will not only allow astronauts to receive appropriate support but also foster an environment where prevention and early intervention are possible.

Before the mission, astronauts already undergo rigorous psychological evaluations, but future long-duration expeditions may require more comprehensive screening using validated tools adapted for spaceflight to better detect vulnerabilities. Existing clinical instruments, such as those used to identify alcohol misuse or psychological distress, could be tailored to this context. Pre-mission programs should also emphasize psychoeducation, covering stress management, sleep hygiene, and the risks of pharmacological dependence, while equipping astronauts with resilience-building strategies such as mindfulness, relaxation techniques, and cognitive-behavioral approaches. Importantly, the responsibility should not rest solely on astronauts; careful pre-selection of pharmaceuticals, prioritizing medications with a lower risk of dependence, is essential to ensure both safety and sustainability during extended missions.

During the mission, the maintenance of psychological wellbeing is determined by a combination of vigilant oversight and the availability of accessible support resources. Asynchronous telehealth has the potential to provide astronauts with professional guidance despite communication delays. Pre-programmed psychological modules and autonomous behavioral health tools, such as VR or AI-driven companions, may help deliver stress management and cognitive-behavioral strategies in real time (Gatti et al., 2022). Wearable devices capable of tracking sleep, stress, and emotional state can support the early detection of difficulties, allowing for timely intervention before problems escalate (Wang et al., 2025). To prevent reliance or misuse while ensuring access to critical medication, tight drug management remains crucial, with limited pharmaceutical stocks being closely monitored and their usage being directed by explicit procedures. On the ISS, pharmaceutical management follows strict protocols to minimize risks of misuse, interactions, or adverse effects in the isolated environment (Wotring and Smith, 2019). Medication supplies are limited, pre-packaged, and inventoried prior to launch. Astronauts do not have free access to medications. All drugs are stored in an onboard medical kit, with usage logged and tightly controlled. The Crew Medical Officer, typically a trained astronaut, assists with medication administration and does not independently prescribe treatments (NASA, 2023c). The ultimate medical authority is the flight surgeon on the ground, who provides guidance through telecommunication and decides when and if medication should be used (Wotring, 2015; Blue et al., 2019).

After the mission, structured psychological follow-up is essential to support reintegration and reduce the risk of maladaptive coping strategies. Systematic screening for depression, anxiety, and substance use disorders should become standard practice, informed by historical accounts of astronauts who faced these challenges upon return. Long-term monitoring, similar to protocols physical health, can help detect psychological difficulties and provide interventions.

Despite rigorous psychological screening and strict monitoring, uncertainties remain regarding cultural and historical variations in substance use. Astronauts are carefully selected and trained, yet the unique stressors of deep space place them in conditions humanity has never faced. In this context, the use of pharmacological support, though often necessary, raises difficult ethical questions. Should psychoactive medications be available to manage stress, anxiety, depression, or sleep? Where is the boundary between treatment and enhancement? How should substance misuse be defined in an environment that reshapes our very understanding of health and human resilience? These questions illustrate the broader challenge of maintaining astronaut health through LM: each pillar brings its own constraints, but together they raise the need for integrated, system-level approaches.

### 4.7 Interdependence of lifestyle medicine pillars in astronaut health

A core principle of LM is that its six pillars function as an interdependent system rather than isolated domains (Frates, 2022). This interdependence is likely amplified in astronaut health, where stressors such as circadian misalignment, microgravity, confinement, and operational workload generate cascading physiological and psychological effects (Yin et al., 2023). For example, on Earth, sleep restriction increases sympathetic activation and cortisol levels (Rogerson et al., 2024), which can impair emotional regulation and increase energy intake by exacerbating appetite and altering metabolic signaling (Broussard et al., 2016), while reduced mood and motivation may decrease adherence to structured exercise protocols (Helgadóttir et al., 2018). Conversely, targeted exercise has been shown to improve sleep efficiency (Zhou et al., 2025), stabilize mood (Zhou et al., 2025), and support immune function (Khune et al., 2024; Ismail et al., 2025), highlighting the synergistic potential of integrated countermeasures. Recognizing the bidirectional interactions among LM pillars (e.g., poor sleep negatively impacts stress, which in turn might influence behaviours and nutritional choices) is therefore essential for future space mission planning and suggests that combined, rather than single-pillar interventions, may offer superior protection for long-duration exploration missions.

## 5 Discussion

This narrative review explored how lifestyle medicine (LM) can guide strategies to optimize astronaut health across mission phases while informing care in remote and underserved Earth settings. To that end, we brought together experts in each field (pillar) and synthesized the relevant literature. Multiple factors constrain nutrition in space, including appetite suppression (Laurens et al., 2019), gut balance disruption (Bergouignan et al., 2016) which could play an important role in mental health (i.e., anxiety and depression) (Xiong et al., 2023), and micronutrient loss (Cooper et al., 2017). These factors highlight the need for sustainable food systems and multiphase strategies across pre-flight, in-flight, and post-flight care. These strategies have potential for remote settings: shelf-stable meals and modular cultivation can improve food security in disaster zones or isolated communities, while astronaut-developed nutrition sensors may support monitoring in low-resource healthcare. The potential benefits of specific probiotics for the gut microbiota and mental health on Earth have generated significant interest in research (Xiong et al., 2023). A plethora of additional studies have demonstrated that prebiotics and postbiotics may have a role in the treatment of mental disorders, such as depression and anxiety (Xiong et al., 2023; Xiong et al., 2022; Munawar et al., 2022; Aslam et al., 2020). Since adequate nutrition alone cannot prevent the effects of microgravity, physical activity becomes a central countermeasure. Physical activity is compromised by microgravity-induced deconditioning (Coupé et al., 2009), requiring pre-flight conditioning (Lambrecht et al., 2017; Petersen et al., 2016), in-flight exercise with specialized devices (NASA, 2024b), and emerging countermeasures such as BFR (Chua et al., 2022; Wang X. et al., 2023) and compact systems (Canadian Space Agency, 2023). Sleep is disrupted by circadian misalignment (Flynn-Evans EE. et al., 2016; Guo et al., 2014; Zong et al., 2025), making circadian alignment, light therapy (Whitson et al., 1995; Czeisler et al., 1991), optimized schedules (Williamson et al., 2011), and structured recovery protocols essential, with pharmacological aids (Barger et al., 2014) used only as adjuncts. Stress management is critical in the face of isolation, confinement, and operational risks (Oluwafemi et al., 2021; Kanas and Manzey, 2008), with countermeasures ranging from cognitive-behavioral (Nakao et al., 2021), listening to music (Khalfa et al., 2003), and biofeedback approaches (Kudo et al., 2014). Social connection is strained by separation (Johnson et al., 2012), crew tensions (Bell et al., 2019), underscoring the importance of team selection, structured communication, and post-flight reintegration support. Finally, while alcohol use is prohibited during space missions by NASA (STD NASA, 2022), the reliance on hypnotics to sleep (Barger et al., 2014) and stimulants underscores the need for strict medication stewardship and long-term monitoring. Taken together, these findings illustrate the value of LM as a holistic framework to sustain astronaut health before, during, and after missions, with lessons transferable to Earth.

However, the current evidence base remains limited by several methodological limitations. Most spaceflight studies involve small sample sizes, absence of control groups, short mission durations, and heterogeneous protocols, making it difficult to generalize findings to future deep-space expeditions (Hardy et al., 2025). Results from Earth-based analogs such as bed rest studies, Antarctic stations, and an underwater habitat, only partially replicate the combined effects of microgravity, radiation, and communication delays, creating a translational gap between simulation and real spaceflight conditions (Cromwell et al., 2021). In addition, available countermeasure studies often evaluate single-pillar interventions in isolation, and few have compared the effectiveness of integrated approaches. Addressing these limitations will be essential to determine which LM-based strategies are truly effective for long-duration missions.

To support this synthesis, Table 1 outlines the principal constraints associated with each LM pillar, along with existing countermeasures and future research priorities.

**TABLE 1:** Summary of the main constraints, mitigation strategies, and future research priorities for space exploration related to each LM pillar.

Pillars	Constraints in space	Identified countermeasures
Nutrition	• Disruption of gut microbiota and electrolyte balance (Bergouignan et al., 2016) • Absence of refrigeration and limited cooking options (Evert et al., 1992) • Long-term storage requirements (3–5 years) reduce nutrient retention and palatability (Cooper et al., 2011) • Micronutrient loss (e.g., potassium, calcium, vitamin D, vitamin K) before and during storage (Cooper et al., 2017) • Appetite suppression in microgravity, altered taste/smell perception, leading to insufficient caloric intake that can be dangerous for long-term missions (Laurens et al., 2019) • Energy imbalance due to a mismatch between intake and expenditure (Bourdier et al., 2022)	Pre-flight: Sensory and nutritional evaluation of space food (Zwart et al., 2009). No specific guidelines (Morrison et al., 2021) In-flight: High-protein intake via enriched food or supplements (Gao and Chilibeck, 2020), closed-loop bioregenerative food systems (Häuplik-Meusburger et al., 2011; Zabel et al., 2016), plant cultivation (Häuplik-Meusburger et al., 2011) Post-flight: Nutrition integrated with physical rehabilitation (Backx et al., 2017; Backx et al., 2018; Wu et al., 2021), and learning lessons from geriatric care, given the similarities (Mulavara et al., 2018; Strollo et al., 2018; Vernikos and Schneider, 2010). No specific guidelines (Morrison et al., 2021)
Physical activity and exercise	• Microgravity-induced muscle atrophy (Bailey et al., 2018; Oganov et al., 1991; Puglia et al., 2018), bone decalcification (Moosavi et al., 2021; Nguyen et al., 2021; Smith et al., 2005), increased joint stiffness (Lambertz et al., 2001), and cardiovascular deconditioning (Coupé et al., 2009) • Muscle atrophy has been observed on missions as short as 9 days (Akima et al., 2000) • Astronauts cannot always have direct access to training equipment (Loehr et al., 2015) • Significant deficits in manual dexterity, dual-tasking, motion perception, vehicle operation ability, and often lower back pain (Lambrecht et al., 2017; Moore et al., 2019)	Pre-flight: Individually tailored training programs according to their specific duties and available time. Focus on building muscle mass, strength, and aerobic capacity. Education on equipment use and posture (Lambrecht et al., 2017; Petersen et al., 2016), and tele-training platforms for remote supervision (Suso-Martí et al., 2021) In-flight: Use of ARED, treadmill, CEVIS (NASA, 2024b), gradual shift from cardiovascular to resistance focus (Petersen et al., 2016) potential integration of compact flywheel (Canadian Space Agency, 2023) devices and BFR cuffs Post-flight: Reconditioning using BFR, training to restore any residual performance loss that degraded due to microgravity (Lambrecht et al., 2017; Petersen et al., 2017) tele-rehabilitation for continued monitoring (Suso-Martí et al., 2021)
Sleep	• Altered light–dark cycles, isolation, and microgravity cause circadian disruption (Flynn-Evans et al., 2016a; Guo et al., 2014; Zong et al., 2025) • Downstream effects on clock gene expression impair internal timekeeping mechanisms (Monk et al., 1998) • Ambient lighting intensity is lower than terrestrial levels (NASA, 2023a) • Reduced total sleep time, altered circadian rhythm, fragmentation, altered architecture (Santy et al., 1988; Putcha et al., 1999) • 45% of administered drugs are intended for sleep disturbances (Putcha et al., 1999) • 70% of astronauts on board space shuttles and ISS used sleep aids (Barger et al., 2014)	Pre-flight: Light therapy to minimize sleep loss and shift circadian rhythm (Whitson et al., 1995; Czeisler et al., 1991) In-flight: Dynamic lighting with short wavelength enriched light when indicated (Barger et al., 2012), strategy optimized schedules (Williamson et al., 2011), judicious use of sleep aids (Barger et al., 2014) and stimulants with clear protocols and medical oversight Post-flight: Recovery protocols using light therapy and continued monitoring. Further research is needed
Stress	• Multiple concurrent stressors such as microgravity, isolation, confinement, noise, and circadian rhythm disturbances (Oluwafemi et al., 2021; Kanas and Manzey, 2008) • Stress induces changes that can be functional or structural (McEwen, 1999) and cause cognitive disorders (Sandi, 2013) • Chronic stress can lead to health problems (Selye, 1978) and performance issues, including difficulty concentrating (LeBlanc, 2009; Chu et al., 2025), impaired memory (Gould and Tanapat, 1999), chronic fatigue (Chu et al., 2025), and poor ability to cope with stressful events (McEwen, 2007) • Stress response varies widely amongst individuals (Selye, 1975; Yaribeygi et al., 2017; Schneiderman et al., 2005) • Complex tasks carried out by astronauts and different management styles can lead to stress (Oluwafemi et al., 2021; Kraft et al., 2003; Del Giudice et al., 2011)	Pre-flight: Selection process emphasizing on personality, intercultural and interpersonal skills, and performance in simulations and interviews, which assess how well candidates manage stress. Psychological and emotional training (Hodzic et al., 2015) In-flight: External monitoring using smart objects such as smartwatches (Frazier and Parker, 2019; Dorsey et al., 2022; Crosswell and Lockwood, 2020), mindfulness and relaxation practices (Pagnini, 2024), personal leisure-time activities, carrying personal items on board (Burattini et al., 2014), and cognitive-behavioral-based methods (Nakao et al., 2021) Post-flight: Counseling and debriefings further research is needed (Kanas, 2015)
Social connection	• Limited privacy, extreme isolation, and confinement create social monotony (Le Roy et al., 2023), crew tensions, and interpersonal conflicts (Bell et al., 2019) • Prolonged separation from family and friends impedes astronauts’ access to key sources of social support (Johnson et al., 2012) • Potential reduced perceived support from crewmates during long missions	Pre-flight: Team-building activities, training in self-care, communication, team care, teamwork and small-group living skills (Landon et al., 2018) In-flight: Videoconferences and phone calls with family, delivery of care packages and fortnightly psychological support sessions (Sipes et al., 2019). For longer missions, consider using AI social robots or VR tools (Gatti et al., 2022) Post-flight: Sustained support to astronauts and their families (Yardley et al., 2021)
Avoidance of risky substances	• Isolation disrupted circadian rhythms, and mission-related stress are known to affect mental wellbeing (Kanas, 1998; Flynn, 2005; Kalb and Solomon, 2007; Kandarpa et al., 2019; Marazziti et al., 2022) • The deterioration of other lifestyle pillars may lead to increased reliance on pharmacological support, as these pillars are closely linked to mental and physical health • Use of hypnotics to manage sleep disturbances, which carries a high risk of dependence (Barger et al., 2014; Petursson and Lader, 1981; Terzano et al., 2003)	Pre-flight: Rigorous psychological screening with clinically validated tools adapted for space, as well as psychoeducation on sleep, stress, and risk of dependence. Medications with a lower risk of dependence should be prioritized In-flight: Tight medication stewardship, limited stocks and access control (Wotring, 2015; Blue et al., 2019), remote health monitoring (HSS, 2025), VR and AI companions (Gatti et al., 2022) Post-flight: Structured psychological follow-up and screening for depression, anxiety, and substance use disorders, destigmatized access to care for astronauts and families

Pillars	Future research priorities
Nutrition	• Address the lack of standardized nutritional guidelines specific to long-duration missions • Define mission phase-specific macronutrient and micronutrient requirements (pre-flight, in-flight, post-flight), including minimum effective intake thresholds • Clarify how nutritional decline influences musculoskeletal loss and immune dysregulation and microbiome changes in microgravity and analogs • Determine how stress, circadian disruption, and sleep loss influence appetite regulation, taste/smell changes, and energy balance in microgravity • Evaluate integrated countermeasures (e.g., nutrition + exercise + sleep + stress management) versus single-pillar strategies • Conduct long-term studies on bioregenerative food systems and plant-based food production systems for safety, nutrient stability, and feasibility beyond low Earth orbit • Determine nutritional strategies to preserve metabolic flexibility and prevent metabolic inflexibility during long-duration missions • Validate microbiome-targeted interventions in analogs and post-flight recovery
Physical activity and exercise	• Determine the minimum effective dose (frequency, intensity, duration) of resistance and aerobic training required to prevent muscle and bone loss and maintain cardiovascular function on long-duration missions • Compare integrated countermeasures versus exercise alone on multisystem outcomes (bone, muscle, cardiovascular, cognitive, immune) • Evaluate the feasibility and efficacy of compact, low-mass exercise technologies (e.g., flywheel devices, BFR training) for missions with limited habitat volume (e.g., Orion or lunar surface operations) • Conduct longitudinal studies to assess individual variability in musculoskeletal and cardiovascular deconditioning and identify predictors for personalized exercise prescriptions • Investigate how reduced physical activity interacts with sleep, cognitive performance, and stress resilience in microgravity and analog environments • Develop personalized exercise prescriptions using wearable-derived physiological feedback to dynamically adjust training intensity and loading across mission phases • Develop evidence-based post-flight rehabilitation protocols, including remote and tele-rehabilitation strategies, to optimize recovery and long-term functional outcomes
Sleep	• Determine minimum sleep duration and quality thresholds required to maintain cognitive and operational performance during long-duration missions • Identify optimal light-based protocols (timing, wavelength, intensity) for circadian entrainment beyond low Earth orbit, including Mars-day adaptation • Evaluate the effectiveness of integrated countermeasures compared with single-pillar approaches • Conduct longitudinal studies in analog environments to assess long-term impacts of circadian disruption on neurocognitive and physiological outcomes • Establish safety, efficacy, and dosing guidelines for pharmacological sleep aids and stimulants during prolonged missions, including risks of dependence • Develop and validate autonomous, non-pharmacological sleep support systems (e.g., dynamic lighting, behavioral interventions, and wearable-based monitoring) suitable for limited-bandwidth environments
Stress	• Address the lack of studies assessing combined LM interventions (e.g., mindfulness + nutrition + sleep stabilization) • Expand longitudinal psychological data in spaceflight cohorts and address small sample sizes • Develop and validate multimodal, wearable-based stress monitoring tools (behavioral and physiological biomarkers) • Test multi-pillar stress reduction packages (e.g., physical activity + mindfulness + sleep stabilization) versus single-technique interventions in analogs and where feasible, in-flight • Evaluate AI-supported psychological countermeasures (e.g., digital CBT, conversational agents) and define guidelines for safe, autonomous use under communication delays
Social connection	• Clarify how declining cohesion contributes to sleep disturbance, stress-related neuroendocrine changes, and downstream health behaviors • Address the limited data on team-level interventions to prevent interpersonal deterioration • Study social cohesion and health outcomes over long-duration missions • Evaluation of VR/AI-mediated social support for Mars-class missions • Characterize protective factors (e.g., shared mission purpose, leadership communication norms) that buffer social stress and support other LM pillars • Develop and evaluate post-flight reintegration strategies, which remain understudied • Evaluate strategies to maintain cohesion and emotional support under progressive communication delays and increasing crew autonomy
Avoidance of risky substances	• Address the lack of research on risk trajectories under chronic isolation and circadian disruption • Develop medication stewardship protocols (e.g., for hypnotics, anxiolytics, stimulants) across long-duration missions, including deprescribing strategies • Develop ethical frameworks for the use of psychoactive medications in conditions of extreme confinement and limited medical backup • Expand evidence on non-pharmacological substitutes for sleep and anxiety management • Examine how deterioration across multiple pillars increases vulnerability to maladaptive coping and substance-related problems • Develop standardized post-flight screening and long-term follow-up protocols for substance-related disorders and psychological vulnerability

LM is well established on Earth as an evidence-based framework for preventing, managing, and even reversing chronic disease through interventions targeting nutrition, physical activity, sleep, stress, social connection, and risky substances. Numerous studies have demonstrated its benefits for cardiometabolic health, mental wellbeing, immune function, and overall longevity, with effect sizes comparable to or greater than pharmacological treatments in some conditions. Translating these benefits to the space environment provides a unique opportunity. LM emphasizes proactive, non-pharmacological, and sustainable strategies that can mitigate physiological decline, reduce reliance on limited pharmaceutical supplies, and enhance resilience. In this context, LM can serve as a preventive health model that prepares astronauts before flight, sustains their physical and psychological performance during missions, and supports recovery and reintegration post-flight. Beyond its application to astronaut health, embedding LM principles in space exploration illustrates the adaptability of this holistic approach to extreme environments, while simultaneously reinforcing its value for populations in remote or resource-limited Earth settings. The six pillars of LM should not be viewed as separate entities but rather as components of an interconnected system. Each pillar influences the others. These interdependencies highlight the importance of approaching LM as a unified framework rather than as separate interventions.

Current astronaut health strategies primarily rely on biomedical countermeasures, including specialized exercise protocols, pharmacological aids, and technological monitoring to address the physiological and psychological challenges of spaceflight. While these approaches are effective in mitigating acute risks, they are often reactive and centered on curative intervention. LM complements and broadens this paradigm by providing a proactive, first-line strategy that emphasizes prevention, resilience, and long-term health maintenance across all mission phases. Rather than replacing curative medicine, LM integrates with it, ensuring that astronauts benefit from both nonpharmacological preventive measures and access to targeted medical treatment when needed. This combined approach offers a more comprehensive and sustainable model for astronaut health, bridging preventive care and curative medicine in the unique context of space exploration.

Emerging technologies offer unprecedented opportunities to strengthen LM in space by enabling both preventive and curative approaches. Wearable sensors can continuously monitor nutrition (energy intake, hydration), physical activity (exercise intensity, muscle loading), and sleep (duration circadian alignment), while also tracking stress responses through heart rate variability, cortisol process, and electrodermal activity. Digital platforms, including artificial AI companions and VR, can enhance social connection and provide behavioral support for stress management, relaxation, or guided exercise. Integrated biomedical monitoring systems, such as real-time ECG analysis for arrhythmia detection (as demonstrated in recent work by Mani et al., 2024), illustrate how LM-oriented technologies can also serve as early-warning tools for acute conditions requiring curative intervention. Moreover, the implementation of human autonomy teaming (HAT) has a strong potential to support astronauts during space missions. HAT refers to the collaborative work of humans and autonomous agent operating interdependently towards a shared goal (O’Neill et al., 2022). This approach is already being deployed on the ISS, where the astronaut AI assistant CIMON-2 supports crew members in their workload, maintenance and repair tasks (Muscles, 2025; Hagemann et al., 2023). In the context of LM in space, HAT can help astronauts to flag a high level of stress, for example, or abnormal physiological data that might suggest an underlying issue. This can prompt the astronaut to do guided meditation exercise and adjust ambient lighting. To operate safely and reliably, HAT agents require robust training on representative scenarios. Public dataset, including high mental workload or stress-induction dataset (e.g., SPACE dataset and WESAD) (Giguère et al., 2025; Schmidt et al., 2018) can be used to train HAT agents. Furthermore, autonomous agents can reduce stress and improve wellbeing as well as contribute to more effective teamwork (Linhardt et al., 2025). In long duration missions or deep-space missions where communication delays make real-time medical supervision impossible, HAT can enhance crew medical autonomy by detecting early physiological anomalies, prioritizing required actions, guiding astronauts through diagnostic or therapeutic procedures, and coordinating multiple autonomous systems to simultaneously support environmental control, health monitoring, and operational tasks. Together, these technologies allow for dynamic feedback loops across all six LM pillars, supporting proactive health promotion while also providing timely detection and management of acute medical problems. In this way, technology-enabled LM framework aligns preventive care with the operational demands of space medicine, ensuring both resilience and safety in extreme environments.

This review draws attention to the value of integrating technologies with the principles of LM to monitor and protect astronaut health. While analog environments such as polar stations, submarines, or underground habitats provide useful models, the space environment amplifies stressors in ways that are unparalleled on Earth. Approaches such as predictive modelling and AI can help anticipate risks, support autonomous decision making, and provide personalized guidance during missions. Beyond spaceflight, these innovations also have strong potential for healthcare on Earth, where they can strengthen prevention, promote patient autonomy, and reduce the burden on healthcare systems across diverse settings, from hospitals to home care.

Behavioral countermeasures informed by LM should be considered an essential complement to traditional medical measures. While medications, nutritional supplements, and specialized equipment remain important for astronaut health, lifestyle-based strategies provide an additional layer of prevention that can be implemented before, during, and after missions. These measures foster autonomy and self-regulation in environments where external medical support is limited. LM’s preventive nature makes it valuable for long-duration missions, reducing medical treatment dependency, strengthening resilience, and sustaining health and performance in extreme conditions. To the best of our knowledge, no study has explicitly applied LM as a comprehensive framework in space exploration. Interestingly, ongoing research in other extreme settings, such as firefighters, demonstrates feasibility by applying four of the six pillars (nutrition, physical activity, sleep, and resilience) (Hershey et al., 2023). This illustrates how LM can be modified for use in high-stress and resource-constrained environments. There is a need to use multidisciplinary, integrative approaches in future studies regarding space medicine. While the space environment represents the most extreme test case for applying LM, many of its challenges are mirrored in isolated or resource-limited settings on Earth.

### 5.1 Application to Earth remote areas

The need for medical autonomy is not exclusive to space exploration. It is also highly relevant in terrestrial environments with medically isolated populations, such as remote or underserved communities. These communities face similar healthcare barriers as those encountered in space, albeit in different contexts. Access to basic medications, primary care providers, hospitals, and emergency services is limited or non-existent.

Applying LM principles can move health promotion in remote communities beyond a reactive model focused on illness management toward a proactive model that fosters autonomy, prevention, and sustainability. This requires aligning technological solutions with the pillars of LM and the social determinants of health rather than relying only on infrastructure. While many healthcare systems emphasize improving connectivity, infrastructure, and human resources to manage illness in remote and marginalized populations, inequities persist despite advances in communication technologies, patient wearables, and remote access platforms. Thus, healthcare access alone is insufficient to improve outcomes in these regions, underscoring the need for integrative, prevention-focused strategies grounded in LM. This process involves reconciling holistic and community-driven aspects of healthcare with the rapidly evolving digital health ecosystem. It also aligns with the RISE philosophy (Relevant, Inspirational, Sustainable, Effective), which seeks to make care: 1. Relevant by embedding it into patients’ daily lives and the spaces where behavior change occurs; 2. Inspirational by leveraging peer support and goal setting to help individuals discover their “why”; 3. Sustainable through digital and virtual delivery methods that lower costs while extending reach; and 4. Effective by applying evidence-based behavior change science supported by both professionals and peers (Mauriello and Artz, 2023). Translating this framework into practice requires examining concrete examples of how lifestyle pillars are challenged in remote environments.

When translating this framework into practice, it is clear that each area will have its own unique ecosystem. As such, the needs of patients in terms of addressing the pillars of LM will also be unique. It has been shown that there is a correlation between poor sleep as a potential causative agent for mental health (Fernandez et al., 2024; Grandner and Fernandez, 2021) and other chronic diseases within Indigenous communities (Kader et al., 2024). Similarly, nutritional optimization may be problematic due to lack of access to fresh fruit and vegetables. As an example for remote communities’ support, the Canadian government has attempted solutions in creating research grants and infrastructure support through Nutritional North Canada (Canada C-IR and NA, 2024). In addition, novel technology-based approaches have emerged, such as Food Security and Structures Canada, a privately funded endeavor creating partnerships for greenhouse structures in remote northern communities (Food Security, 2025). Ultimately, whether through circadian misalignment, limited food access, or systemic inequities, all pillars of LM are undermined in remote communities. This underscores the need for innovative countermeasures such as telehealth, which can help overcome geographic and systemic barriers to care. In this context, the CSA has developed the Connected Care Medical Module (C2M2), a container-based medical unit designed to integrate AI tools with advanced medical technologies. These mobile modules are deployed both in remote regions and urban centers across Canada to enhance access to care. The long-term vision for C2M2 extends beyond Earth, with prototypes envisioned for deployment on platforms such as the ISS or the Lunar Gateway as part of future space exploration missions (Canadian Space Agency, 2022).

Beyond physical health, technological solutions aimed at the pillars of stress management and social connection represent another domain worth exploring for both deep space missions and remote rural communities. Research in VR has already demonstrated a promise in stress management and post-traumatic stress disorder (PTSD) recovery. For example, Dr. Skip Rizzo and colleagues (Khune et al., 2024) have pioneered the use of VR to prepare soldiers and first responders for stressful or traumatic encounters, as well as to provide exposure-based therapy for PTSD and other mental health disorders (Rizzo and Shilling, 2017; Rizzo et al., 2025; Rizzo et al., 2024). While there are currently no explicit examples of using VR as a digital health tool for remote communities, we could certainly envision applications of this technology in this domain. Although applications would differ between remote populations and astronauts, VR could serve as a valuable tool to mitigate isolation, support psychological resilience, and complement existing countermeasures in both contexts. For instance, rural Indigenous youth in countries such as Canada, Australia, and the United States experience disproportionately high rates of mental illness and suicide (Pollock et al., 2018), with the United Nations recognizing Indigenous peoples as among the most disadvantaged and vulnerable populations worldwide (DESA, 2025). In this context, culturally adapted VR environments could provide innovative avenues for support by creating virtual spaces where elders and youth interact, fostering intergenerational connection and cultural continuity. Beyond strengthening social ties, VR platforms could also be harnessed to promote healthy behaviors, such as encouraging proper nutrition, integrating exercise through gamified movement, or offering mental health coaching to enhance stress management and sleep hygiene. Digital health technologies offer a promising means of bridging these gaps. However, introducing wearable technologies requires careful consideration of cultural context and data quality (Swahn et al., 2024).

Health, when viewed through the framework of the six pillars, is not limited to treating disease but is fundamentally about prevention and maintaining balance across interconnected domains of wellbeing. This integrated approach becomes even more powerful when combined with technological innovations, offering support for ongoing monitoring and guidance. Taken together, LM provides a promising framework to sustain astronaut health during deep space missions while simultaneously strengthening preventive and primary care strategies on Earth, underscoring its bidirectional translational potential between space and terrestrial contexts. Insights from space medicine, such as innovations in wearable health sensors, telemedicine platforms, and nutritional strategies optimized for microgravity can inform the delivery of care in remote or resource-limited settings on Earth. Conversely, evidence from Earth-based LM interventions, including behavior change strategies, digital health tools, and community-based prevention models, can support the design of more sustainable and human-centered countermeasures for astronaut health.

## 6 Conclusion

LM provides a comprehensive framework that combines preventive strategies with integrative approaches to address the unique vulnerabilities of spaceflight. By considering the pillars as an interconnected system rather than isolated domains, LM complements conventional countermeasures, enhances autonomy, and supports health in environments where external medical support is limited. No study has yet explicitly applied this framework to space exploration, underscoring both the originality and the necessity of this perspective.

The value of LM extends beyond space, offering potential scalable and sustainable solutions that can strengthen primary care-based prevention and to address persistent health inequities in remote and underserved regions on Earth. Viewing astronaut health and terrestrial health through the same lens highlights the bidirectional translational power of countermeasures developed in one context to benefit the other.

Future research should therefore move beyond single-pillar countermeasures and prioritize integrated, longitudinal studies in analog environments to quantify the synergistic effects of combined LM interventions, such as nutrition with mindfulness or exercise with sleep stabilization, versus single-pillar countermeasures. Additional priorities include: 1. defining the minimum effective recommendations of multi-pillar strategies required for long-duration missions; 2. validating wearable and biomarker-based monitoring tools to assess stress, sleep, and immune function in microgravity and isolation; 3. addressing the translational gap between analog studies, short-duration spaceflight, and deep-space missions profiles; and 4. developing personalized, adaptive LM protocols, potentially supported by AI, to respond to dynamic physiological and psychological changes over time. Advancing these research directions will help assess whether the integrated LM framework provides additional benefits beyond isolated countermeasures, thereby informing future mission planning. Such efforts will not only strengthen crew health and mission success but also contribute to more equitable, preventive, and autonomy-centered models of healthcare delivery. Ultimately, this advances a vision of healthcare that is preventive, sustainable, and adaptable across the most extreme frontiers, from space to Earth’s most underserved regions.

## Abbreviations

ACLM, American College of Lifestyle Medicine; ARED, Advanced Resistive Exercise Device; AI, Artificial Intelligence; BFR, Blood Flow Restriction; CELS, Controlled Ecological Life Support System; CEVIS, Cycle Ergometer with Vibration Isolation and Stabilization system; CIMON, Crew Interactive Mobile CompanioN; C2M2, Connected Care Medical Module; CRH, Corticotropin Hormone; CSA, Canadian Space Agency; DORAs, Dual Orexin Receptor Antagonists; ECG, Electrocardiogram; ESA, European Space Agency; HAT, Human Autonomy Teaming; LM, Lifestyle Medicine; LSAH, Lifetime Surveillance of Astronaut Health; IMM, Integrated Medical Model; ISS, International Space Station; NASA, National Aeronautics and Space Administration; PTSD, Post-Traumatic Stress Disorder; RISE, Relevant, Inspirational, Sustainable, Effective; SANRA, A Scale For The Quality Assessment Of Narrative Review Articles; SPACE dataset, Stress Physiological And Cognitive Evaluation dataset; VR, Virtual Reality; WESAD, Wearable Stress and Affect Detection dataset.
