# Correction: Space exploration and lifestyle medicine: a narrative review of its implications for astronaut health and remote Earth-based environments

**DOI:** 10.3389/fphys.2026.1827594

**Published:** 2026-03-20

**Authors:** Raphaëlle Giguère, Alexandre Marois, Daniel Fortin-Guichard, Jonathan Charest, Julie Rocheleau, Keith Thompson, Michael Stolberg, Philippe St-Martin, Audrey Bergouignan, Victor Niaussat, Jean-Sébastien Paquette, Marie-Pierre Gagnon, Maxime Sasseville, Caroline Rhéaume

**Affiliations:** 1 Department of Medicine, Faculty of Medicine, Université Laval, Québec, QC, Canada; 2 VITAM – Research Center on Sustainable Health, Québec, QC, Canada; 3 École de psychologie, Faculté des sciences sociales, Université Laval, Québec, QC, Canada; 4 Department of Kinesiology and Physical Education, Sylvan Adams Sports Science Institute, McGill University, Montréal, QC, Canada; 5 Idorsia Pharmaceuticals Canada, Montréal, QC, Canada; 6 Department of Psychology, Université du Québec à Montréal, Montréal, QC, Canada; 7 Department of Family Medicine, Schulich School of Medicine and Dentistry, Western Space Institute, Western University, London, ON, Canada; 8 Département des sciences de l’activité physique, Université du Québec à Montréal, Montréal, Canada; 9 Faculty of Physical Activity Sciences, Université de Sherbrooke, Sherbrooke, QC, Canada; 10 Research Centre on Aging, Université de Sherbrooke, Sherbrooke, QC, Canada; 11 Pluridisciplinary Hubert Curien Institute (IPHC UMR 7178), French National Center for Scientific Research (CNRS), Université de Strasbourg, Strasbourg, France; 12 Division of Endocrinology, Metabolism and Diabetes, Anschutz Health and Wellness Center, University of Colorado, Aurora, CO, United States; 13 Thales AVS, Mérignac, Nouvelle Aquitaine, France; 14 Primary Care Research and Innovation Laboratory (Laboratoire ARIMED), Groupe de Médecine de Famille Universitaire du Nord de Lanaudière, Joliette, QC, Canada; 15 Faculty of Nursing, Université Laval, Québec, QC, Canada; 16 Quebec Heart and Lung Institute-Research Center (IUCPQ), Québec, QC, Canada

**Keywords:** lifestyle medicine, microgravity, remote Earth-based environments, space exploration, space medicine

An incorrect **Funding** statement was provided. The correct **Funding** statement reads:

“The author(s) declared that financial support was received for this work and/or its publication. RG holds a research grant for her doctoral studies from the VITAM Research Center and Canadian Space Agency.”

The original version of this article has been updated.

